# Developing an integrated emergency medical services in a low-income country like Nepal: a concept paper

**DOI:** 10.1186/s12245-020-0268-1

**Published:** 2020-02-07

**Authors:** Deepak Bhandari, Nabin Krishna Yadav

**Affiliations:** 1 Department of Anesthesia, Critical care and Pain, Nepal Mediciti Hospital, Bhainsepati, Kathmandu, Nepal; 2 Department of Anesthesiology And Critical Care, Chitwan Medical College, Bharatpur-10, Chitwan Nepal

**Keywords:** Emergency medical services, Pre-hospital care, Emergency system, Nepal

## Abstract

**Background:**

The main aim of emergency medical services (EMS) should be to provide universal emergency medical care which is EMS system available to all those who need it. Most of the developed countries have an integrated EMS system that is accessible by a single dial number in the whole country. Nepal does not have a proper EMS system. We conducted a literature review regarding methods of developing an integrated EMS system in Nepal.

**Result:**

The fragmented system, high demand–low supply, inequity with the service, and inadequately trained responders are major problems associated with EMS in Nepal. Nepal too should develop an integrated single dial number EMS system to meet the current demand of EMS. Having a paramedic in ambulances as the first responders will prevent chaos and save critical time. Funding models have to be considered while developing an EMS considering the capital as well as operational cost.

**Conclusion:**

Nepal can develop a public private partnership model of EMS where capital cost is provided by the government and operational cost by other methods. Community-based insurance system looks more feasible in a country like Nepal for generating operational cost.

## Background

Emergency medical services (EMS) means out of hospital acute medical care and/or transportation provided to a patient with  an illness or an injury which the patient or the medical practitioner (doctor, nurse, or a paramedic) believes constitutes a medical emergency. The main aim of EMS should be to provide universal emergency medical care which is EMS system available to all those who need it [1]. EMS system has to deal with a large spectrum of diseases ranging from simple injuries to life-threateningconditions such as septicemia, premature labor, myocardial infarction, polytrauma  etc. The overall burden to EMS system is hard to define and measure. The rapid epidemiological transition from communicable disease to non-communicable disease has been seen in the last 20 years not just in developed countries but in developing countries as well [2]. In this time period, there has been an overall rise in demand for EMS [3]. An increase in demand has also increased financial burden creating a big challenge to cover the cost [4].

It is believed that an effective EMS system should be able to provide EMS to all the people who need it [1]. With more than half of the global population living in urban areas, demand for EMS has increased significantly even in developing and underdeveloped countries [4]. Most of the developed countries have an integrated EMS system that is accessible by a single dial number in the whole country. Developed countries have improved their trauma mortality rate which has been credited to increase in pre-hospital care and trauma prevention strategies [5]. Injury is now the leading cause of death in low- and middle-income countries which reflect a high necessity of a proper EMS system in these countries [1]. EMS infrastructure in developing countries is underdeveloped and lack of roads, and transportation vehicles have added more to the problem [6]. A lack of proper EMS system means a higher number of deaths which could have been prevented easily.

### Overview of EMS in Nepal

Nepal has an EMS model which can be referred to as “The neglect model” [5] where no rules and regulations exist about service providers. Pre-hospital care in Nepal is very minimal, and EMS system is still a new concept in Nepal. In a research done by Gongal et al. [7] in an emergency room of Patan hospital located in Kathmandu, only 9.9% patients arrived in ambulance, whereas 53.6% came in a taxi, 11.4% came by private vehicle, 13.5% came by bus, 5.4% came by bike, and the rest 6.2% came by other modes of transportation. Police are always the first person to be asked for help in case of road traffic accidents. Ambulance services are operated by not just government but multiple trusts, non-profitable organizations, and also almost all private hospitals. Most of the ambulances have no formal paramedics and are not able to accommodate any medical equipment [8]. These ambulances carry pediatrics, adult patients, and even those who require a ventilator. The general population lacks proper knowledge and information about hospitals and health care. Most of the time patients land up in the wrong hospital where the service for a particular disease or condition is not available. They are further referred to another hospital causing loss of critical time period increasing morbidity and mortality of the patient. Most of the private hospitals have their own ambulance and are also responsible for their function and maintenance [8]. The patient is charged by these hospitals for their use of services. Charge usually depends on the amount of distance covered which is similar or more than the cab service.

A few years ago, Nepal was successful in establishing its first organized ambulance service system named Nepal Ambulance Service (NAS) [7]. NAS is a non-profitable private organization. It officially began its service of pre-hospital emergency care in 2011 AD. NAS has a toll free number 102 and started its operation in greater Kathmandu and Patan and later extended its operation to Pokhara and Chitwan districts. In Kathmandu district, it started with 5 ambulances and now has around 40 ambulances. It provides pre-hospital medical care by emergency medical technicians (EMTs) who undergo 3 months training by Stanford University School of Medicine (USA) experts from Stanford Emergency Medicine International (SEMI). So far, there are three trainings held that have trained 27 EMTs. These EMTs provide variety of medical interventions including BLS, ALS, splinting fractures, bracing spinal cord injuries bleeding control, airway management, and starting IV fluids for patients in shock. This service is simply insufficient and occupies a very small market share. Although it is in the early phase, it has a very good potential to expand and provide service to larger range of population.

Number of calls to Nepal Ambulance service per year

**Table:** 

May to April	Number of calls
2011–2012	2093
2012–2013	3963
2013–2014	3715
2014–2015	2761
2015–2016	3517
2016–2017	4232
2017–2018	5480
2018–2019	5198
2019 May till September	2783
Total	33,742

Total cases—33,742, free cases—33,585, delivery cases—48, mass casualty cases—109

Nepal government has waived taxes while purchasing and running the ambulance to ease the EMS system in the country. Nepal also has helicopter-based emergency services (HEMS) throughout the entire country. HEMS have proven to be a great asset in a mountainous country like Nepal. Few private airline companies have been providing these services with great efficiency in recent days. Lack of medical personnel and equipment has reduced its efficiency below its potency. Also, its charge are high and beyond the reach of the average Nepalese population. The high amount of tourist traveling especially in the Annapurna and Everest region has lead to quite a good number of research and advancement in high altitude medicine. Lately, several efforts have been made to train a doctor in mountain medicine and rescuing the trekkers [8]. The government of Nepal has recently initiated a program to provide free HEMS for pregnant and laboring women during an emergency in remote parts of Nepal. Although HEMS have provided a great benefit, it lacks a proper system and multiple private companies operating on their own have added complexity to an already chaotic system. It has been developed and runs as a business rather than providing service to the patients.

The fragmented system, high demand–low supply, inequity with the service, and low quality of the responders are major problems associated with EMS in Nepal. Although HEMS rescue has boosted the system, Nepal still does not have its own proper air ambulance which is more spacious than a helicopter and can accommodate more people required for rescue. A research conducted by Taylor et al. [9] in Australia which has been using HEMS for more than 20 years concluded that HEMS often resulted in over triage leading to higher financial burden and workload at trauma centers. Authors of this paper [9] also say patients with minor injuries make the majority of transport accounting for unnecessary larger funding requirements. HEMS are expensive and its misuse can have a great deal of financial burden to a poor country like Nepal.

### Developing a single number EMS system in Nepal

The basic principle of having EMS system worldwide is to have a common emergency communication number connected to responsive agencies [10]. To fulfill the aim of accessible universal emergency medical care, Nepal too should develop an integrated single dial number EMS system. As new skills, technology, and interventions continue to evolve, the cost of providing pre-hospital care is rising rapidly which makes funding for EMS a bigger issue [11]. Determining the cost of EMS services and having an appropriate level of funding will help an organization to optimize patient outcomes and resources [11]. Just like EMS systems, funding methods and approaches also vary from place to place. In the USA itself, there are multiple ways of funding EMS depending on the local system and the federal “medicare” rules [12]. At some places, EMS are funded by local tax income and then the individual or insurance companies are charged directly for the service, at others, local government authorities may have a contract with private companies. Funding of an EMS system needs to cover both capital and operational cost.

Taxes are the most common and widely used method by all countries to fund EMS system. New taxation rules can also be introduced by a city or government which can be specifically marked for “EMS” as a source of funding. In 2009, in New Mexico, USA, a legislature was introduced that increased liquor excise tax with the aim of improving the delivery of EMS system. Private foundations and corporate programs donate a large amount of funding for various sectors such as education, arts, and community development every year. Such donations can be used for EMS. To create an EMS system in Jaffna, Sri Lanka (previously a war zone area), a grant was provided by a US Office of Foreign Disaster Assistance and a US NGO named “Ameri Cares” for capital funding [13]. An additional source for funding was then diverted from an existing health fund, and a nominal fee is charged to the user [13]. The same approach can be implemented to develop EMS system in Nepal. Traditional bank loans or soft loans can be used as a source for capital setup, and other measures can be used to derive the fund for operational cost. Cities and governments can borrow the fund required for the capital setup cost. The government-owned ambulance and hospital were upgraded in Jaffna, Sri Lanka, to set up a new system of EMS instead of implementing a new service [13]. A huge amount of money was saved in this manner. Similarly, to establish a EMS system in Punjab, Pakistan, local resources were used to build the infrastructure for ambulance stations and ambulance which saved foreign exchange of around US$ 25.5 million [14]. A low-cost efficient model of EMS was developed in Pakistan with high use of local manpower and manufacturing units [14].

Many countries in the world such as Japan, Scandinavian countries, and Canada fund the operational cost for EMS via the taxation system. In a few parts of the world, even tourists have an obligation to pay the tax referring to it as a “transient occupancy tax” or “bed tax” for emergency services. Local authorities can provide a partial amount of money to run EMS system which is widely practiced in many parts of the world [12]. In 2003, as part of community funding, Queensland introduced a different method for funding EMS naming it as “Community Ambulance Cover” [15]. Each resident had to pay a levy surcharge in their electricity bill which was then used to fund EMS system [15]. This provision provided a “free” ambulance service to the residents of Queensland [15]. A public fund system was developed in Jaffna, Sri Lanka, with each person contributing a small sum of money annually to cover the deficit fund for operating EMS system [13]. The general practice in EMS has been to award a contract to the private EMS company by local government authority for a limited period of time which ensures a check and balance system for performance requirements [4]. In India, the **“**Dial 108” service present in 14 states is operated under a PPP model. PPP system operating in a few states of India also works on the same basis with the government providing the financial aid, and private companies providing service without any charge. Directly charged persons or insurance companies for the service is an easy way to gain funds for EMS. Various types of fines and penalties can be incorporated as a measure to raise funds for the EMS. Fines collected by traffic are an easy way to generate funding for the operational cost. Membership fees are a form of private insurance where individuals can choose to join the service. Once a member, they may get the service in free or with a certain amount of co-payment.

The concept of insurance and copayment is a relatively newer term in Nepal. Few insurance companies provide insurance to healthcare facilities. There is no clear rule and policies for EMS coverage by these companies. Having multiple insurance coverage in the market creates competition. Competition for lower prices and different kinds of insurance coverage systems among multiple providers is thought to increase access to health care for consumers in a lower transaction rate [16]. Also, another thought is having insurance may significantly increase the number of emergency department visits by low severity patients leading to misuse of ambulance service by people who do not require it [4]. Co–payment has always been a controversial issue in the health care market [4]. Sharing for ambulance cost is believed to lower the demand for EMS which can reduce government cost [17]. Overall studies show that the co-payment system in emergency services is seen as a safe way to share the cost and reduce unnecessary visits to emergency departments.

So, from the literature, we can say that public insurance would increase the access of EMS for people with socioeconomic disadvantages who require it more while private insurance can have a fair amount of “moral hazard”. A flexible reimbursement policy to pay EMS provider could reduce the “moral hazard”. Nepal lacks a proper health insurance system and laws regarding it. There are very minimal health insurance and its use. Insurance also does not cover EMS in it. Developing a public insurance model would provide a larger benefit to people.

### Opportunity cost

A big question arises, is it wise for a developing country to divert its already scarce money in EMS systems? Additional expenditures will be required for creating a proper health system that incorporates EMS such as emergency departments, well-equipped hospitals with more beds, doctors, and nurses. The integration of all the medical services may result to be more costly and treat fewer people compared with a public health intervention. Lack of health insurance in Nepal makes this problem even worse. Providing a free EMS is complex as defining what is a medical emergency and from whose point of view is not easy [18]. The use of ambulance service by non-urgent patients would result in hospital overcrowding, limit ambulance response to patients who require it, and be a waste of resources. This would have been a waste of resources as the same money could be used for other health care services which would have saved more lives. Thus, it would be wise for the Nepalese government to conduct a cost-benefit analysis before establishing an integrated EMS system.

## Conclusion

Demand for EMS has increased worldwide including the developing nations such as Nepal. This is likely to rise further because of the epidemiological transition. Therefore, countries like Nepal should be prepared to develop the EMS system. Funding models have to be considered while developing an EMS considering the capital as well as the operational cost. A debatable question is how should it be funded—publicly/privately or public-private partnership. Since the socioeconomic conditions of people are different in different countries, it is vital for a country like Nepal to develop EMS system in a private-public partnership model. It can develop an EMS where capital cost is provided by the government and operational cost by other methods. We also suggest that each ambulance has its own qualified paramedic person who would do the triage for the patient. Funding for operating EMS can be supported by insurance, co-payment, or both. Insurance may be private or community-based (i.e., universal as described above). Insurance has advantages like it reduces catastrophic expenditure and provides access more easily and to a larger population. Major disadvantage for Nepal is that the majority of its people do not have any kind of insurance. Community-based insurance system looks more feasible in a country like Nepal. Universal insurance schemes look to be more useful for Nepal but will add up the higher financial burden to the government. Lower co-payment may result in higher demand for EMS, whereas higher co-payment may restrict low socioeconomic group of people from using the service. So, a high degree of caution should be applied before implementing co-payment. Studies in developed countries show that demand for serious cases of EMS is inelastic to price but study done in a developing country says price does affect the demand especially for those who live in rural areas, are less educated, and have low income.

## Data Availability

Not applicable

## Abbreviations

EMS: Emergency medical services
HEMS: Helicopter-based emergency medical services
NAS: Nepal Ambulance Service
PPP: Public-private partnership
